# Editorial: Sleep and circadian rhythm disruptions associated with substance use disorders

**DOI:** 10.3389/fnins.2023.1165084

**Published:** 2023-04-18

**Authors:** Lais F. Berro, Rodrigo A. España, Jessica A. Mong, Robert W. Gould

**Affiliations:** ^1^Department of Psychiatry and Human Behavior, University of Mississippi Medical Center, Jackson, MS, United States; ^2^Department of Neurobiology and Anatomy, College of Medicine, Drexel University, Philadelphia, PA, United States; ^3^Department of Pharmacology, University of Maryland, Baltimore, MD, United States; ^4^Department of Physiology and Pharmacology, School of Medicine, Wake Forest University, Winston-Salem, NC, United States

**Keywords:** sleep disruptions, circadian rhythm disruptions, substance use disorder (SUD), alcohol use disorder (AUD), preclinical model

Individuals with substance use disorder (SUD as well as alcohol use disorder (AUD) report difficulty sleeping, which often persists into extended periods of abstinence and is associated with increased risk of relapse (Angarita et al., 2016; Valentino and Volkow, 2020). Moreover, disrupted sleep and insomnia are known risk factors for the development of SUD/AUD (Dolsen and Harvey, 2017; Roehrs et al., 2021; Troxel et al., 2021). Decades of research have reported neurobiological alterations following chronic licit and illicit drug exposure, many of which regulate sleep, arousal, and circadian rhythms. However, investigations on the neurobiological changes underlying the relationship between sleep/circadian rhythms and drug or alcohol-related behaviors are in their infancy. Thus, interrogating this relationship is integral for understanding risk factors that may increase vulnerability to SUD/AUD, and for improving treatment strategies for SUD/AUD. This focused Research Topic sheds some light on these gaps in knowledge by examining the effects of sleep deprivation on drug-intake or preference, as well as effects of acute or repeated drug exposure on sleep, arousal and/or regulators of circadian rhythms. This collection spans multiple drug classes including opioids, stimulants, and ethanol across species along with multiple time points, including acute and chronic exposure as well as acute and protracted withdrawal.

Eacret et al. describe a set of studies examining how sleep deprivation in mice affects oral morphine consumption and preference using a 2-bottle choice procedure, as well as morphine conditioned place preference. Mice were sleep deprived for the first 8 h of the light (inactive) phase for 3 consecutive days/week for 4 weeks prior to drug exposure and behavioral testing. Sleep deprivation resulted in less oral morphine intake compared to mice who remained undisturbed. No difference in morphine conditioned place preference was observed after sleep deprivation compared to control animals. The authors concluded that recovery sleep after deprivation decreased morphine intake without altering morphine reward.

Bjorness and Greene detail chronic (13 days) effects of three times per day non-contingent administration of cocaine on sleep and waking durations, as well as spectral power, using electroencephalography (EEG) detection in male mice. Repeated cocaine administration increased arousal, supported by increased sleep latency and gamma activity, effects that persisted for 2 weeks into abstinence. Further studies involving 4-h sleep deprivation prior to and following cocaine administration suggested effects were likely due to persistent increases in arousal without affecting homeostatic sleep drive.

Jones et al. examined potential sex differences between male and female rats during acute withdrawal and protracted abstinence following ethanol vapor exposure. Compared to an alcohol naïve state, acute withdrawal led to increased wake duration and reduced rapid eye movement (REM) sleep duration. Sex differences were observed in the time for REM latency to return to normal during protracted abstinence in that females did not return to baseline REM latency values within the 4-week abstinence period. Together, these studies enrich literature demonstrating distinct effects of ethanol withdrawal and protracted abstinence on sleep, specifically detailing sex-dependent differences on sleep macro- and micro-architecture during these phases.

Extending studies to rhesus monkeys, where sleep architecture is more analogous to humans, Berro et al. describe the effects of acute administration of methamphetamine in the morning on sleep later that night. Methamphetamine dose-dependently increased sleep latency and waking time after sleep onset. Sleep architecture was also affected in both duration (decreased slow wave and REM sleep) and percentage, such that sleep shifted to lighter sleep and less restorative slow wave sleep. Further, sleep in N3 and REM stages was reduced at lower doses than those needed to impact total sleep time, suggesting that these stages may be more sensitive to drug-induced sleep disturbances. Subsequently, the night after methamphetamine administration, sleep rebound effects were apparent.

Lastly, Valeri et al. examined post-mortem tissue from humans with SUD, major depressive disorder (MDD) comorbid SUD/MDD or healthy controls to examine possible mechanisms that may contribute to altered sleep/circadian rhythms associated with these conditions. These authors reported altered hippocampal somatostatin signaling molecules and clock gene expression in patients with SUD or MDD, along with changes in amplitude in the diurnal rhythm of gene expression. Together, the authors suggest that these alterations may influence sleep and circadian rhythms, ultimately impacting memory consolidation that may influence risk of relapse.

Preclinical studies afford the opportunity for longitudinal and within-subject assessments to determine the bidirectional relationship between drug exposure and sleep. This research collection highlights several common themes that align with subjective reports in humans. Drug exposure affects sleep and neurobiology and, in many cases, these alterations persist into abstinence. Sex-dependent differences in sleep alterations are apparent in duration and magnitude. This collection adds to a growing literature beginning to interrogate the relationship between SUD/AUD, sleep/circadian regulation, and waking behaviors, and also highlights areas for future research.

With the exception of Eacret et al. the preclinical research included in this collection examined effects of non-contingent drug administration. Prior literature (e.g., Lecca et al., 2007; Orejarena et al., 2009) highlight distinct neurobiological adaptations following non-contingent, experimenter-administered drug vs. contingent drug self-administration. Thus, while non-contingent administration ensures equivalent drug exposure amongst treatment groups, future self-administration procedures are also needed to complement this relatively nascent field. It remains to be determined if multiple drug classes of misuse (e.g., opioids, stimulants, alcohol) alter sleep through convergent mechanisms or, given the multitude of circuits/neurotransmitter systems regulating sleep/circadian processes, distinct neurobiological alterations result in overlapping disturbances. Careful examination of sleep parameters (NREM/REM/spectral characteristics) and sleep control (homeostatic/circadian) may aid such determination. Multiple cutting-edge neuroscience techniques are now being applied to understand circuit and cell-specific mechanisms contributing to regulation of sleep/wake cycles and circadian rhythms in rodent studies. It is equally important to examine drug effects on sleep in higher order species with monophasic sleep cycles (e.g., non-human primates) and to study changes in sleep stages (non-REM sleep stages N1, N2, N3, or REM sleep). Future research needs to build on these studies with a cross-disciplinary approach to broaden our understanding within the context of SUD/AUD, with the goal of incorporating sleep remediation as a therapeutic target to aid SUD/AUD treatment.

## Author contributions

RG wrote the first draft of the manuscript. All authors contributed to manuscript revision, read, and approved the submitted version.
